# The Non-Coding Oncogene: A Case of Missing DNA Evidence?

**DOI:** 10.3389/fgene.2012.00170

**Published:** 2012-09-12

**Authors:** Puja Shahrouki, Erik Larsson

**Affiliations:** ^1^Department of Medical Biochemistry and Cell Biology, Institute of Biomedicine, The Sahlgrenska Academy, University of GothenburgGothenburg, Sweden

**Keywords:** cancer, non-coding RNA, microRNA, lncRNA, lincRNA, T-UCR, mutation, somatic alteration

## Abstract

The evidence that links classical protein-coding proto-oncogenes and tumor suppressors, such as *MYC*, *RAS*, *P53*, and *RB*, to carcinogenesis is indisputable. Multiple lines of proof show how random somatic genomic alteration of such genes (e.g., mutation, deletion, or amplification), followed by selection and clonal expansion, forms the main molecular basis of tumor development. Many important cancer genes were discovered using low-throughput approaches in the pre-genomic era, and this knowledge is today solidified and expanded upon by modern genome-scale methodologies. In several recent studies, non-coding RNAs (ncRNAs), such as microRNAs and long ncRNAs (lncRNAs), have been shown to contribute to tumor development. However, in comparison with coding cancer genes, the genomic (DNA-level) evidence is sparse for ncRNAs. The coding proto-oncogenes and tumor suppressors that we know of today are major molecular hubs in both normal and malignant cells. The search for ncRNAs with tumor driver or suppressor roles therefore holds the additional promise of pinpointing important, biologically active, ncRNAs in a vast and largely uncharacterized non-coding transcriptome. Here, we assess the available DNA-level data that links non-coding genes to tumor development. We further consider historical, methodological, and biological aspects, and discuss future prospects of ncRNAs in cancer.

## Introduction

Already in the late nineteenth century, more than a decade before the term “gene” was coined, it was suggested that somatic chromosomal alterations could form the basis of tumorigenesis (Von Hansemann, 1890). Proof had to wait until after the birth of modern cytogenetics, when the first reported recurrent genomic aberration in cancer, the famous t(9;22) Philadelphia chromosome translocation, was described in the 1960s (Nowell and Hungerford, 1961). With more than half a century’s worth of research since that milestone, great advances into understanding the complex genetic nature of cancer have been made (Hanahan and Weinberg, 2011). It is now firmly established that tumor development depends on genomic instability, acquired genetic variability, and microevolutionary selection (Nowell, 1976). From the discovery of the first somatic mutation of a proto-oncogene in 1982 (Reddy et al., 1982; Tabin et al., 1982) to today, hundreds of genes that are somatically mutated or otherwise genomically altered (amplification, deletion, translocation, or epigenetic modification) have been identified and classified as cancer genes (Futreal et al., 2004). Historically, the main focus has been on coding genes and their protein products, and only recently have non-coding RNAs (ncRNAs) started to gain attention as contributors to the development of cancer.

**Figure 1:**
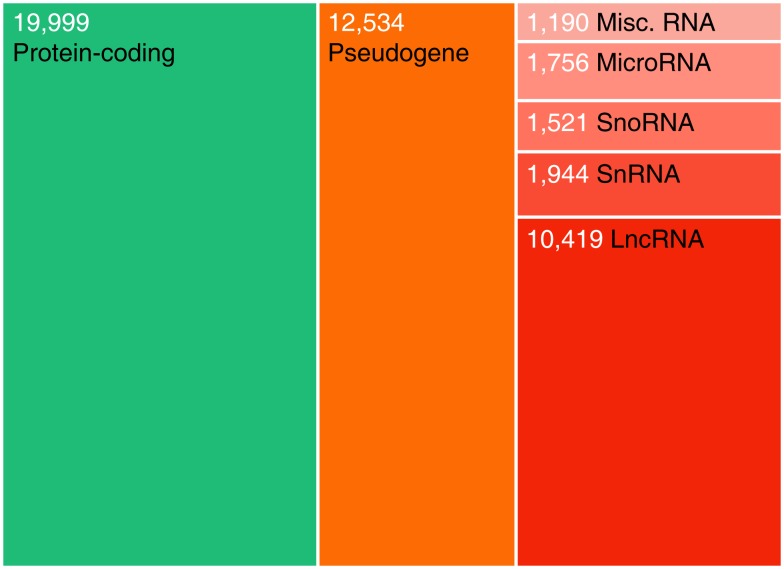
**Relative abundances of major human gene categories**. The figure is based on the GENCODE (Harrow et al., 2006) annotation (version 11), and numbers refer to gene counts rather than transcripts. Note that additional transcribed loci have been described in other high-throughput gene annotation efforts. Coding genes and lncRNAs were defined as described previously (Jeggari et al., 2012). Pseudogenes here refer to the GENCODE “pseudogene” and “polymorphic pseudogene” categories, and do not include ncRNA pseudogenes. “Misc. RNA” mainly comprises Y RNAs and 7SK RNAs.

The classic perception of the genome as mainly a substrate for transcription of protein-coding genes has been significantly revised during recent years. High-throughput RNA profiling has revealed that the genome is pervasively transcribed, and that the number of non-coding genes rivals that of coding genes (Figure 1; Kapranov et al., 2002; Harrow et al., 2006; Birney et al., 2007). The discovery of microRNAs has greatly increased the appreciation for ncRNA beyond classical RNA genes, such as ribosomal and transfer RNAs. In addition to small ncRNAs such as microRNAs, transcriptomic studies have revealed an abundance of long ncRNAs (lncRNAs) that lie interspersed with coding genes in complex ways (Carninci et al., 2005; Katayama et al., 2005; Guttman et al., 2010). These constitute a substantial fraction of all human genes (Figure 1), and greatly complicate our view of the mammalian transcriptome.

As awareness grows that ncRNAs participate in important cellular processes, it is also natural to ask whether alterations in ncRNA activities contribute to tumor development. Cancer is a disease of the genome, and ncRNAs should in principle be susceptible to activation, deactivation, and functional modification through introduction of the same types of somatic genomic aberrations that are known to affect coding cancer genes. The completion of the HUGO project (International Human Genome Sequencing Consortium, 2004), and the advent of high-throughput methods for molecular profiling (e.g., microarrays and next-generation sequencing instruments), has greatly facilitated the study of ncRNAs in cancer. However, despite well over 2000 articles relating to microRNAs in cancer being published in 2011 alone (source: PubMed), evidence of specific somatic alteration at the level of DNA still remains relatively sparse. It is also worth noting that many of the techniques used in the pre-genomic era for finding oncogenes and tumor suppressors, such as cell transformation using tumor DNA fragments followed by molecular cloning, or the study of transforming retroviruses (Weinberg, 1982; Knudson, 1993), are not inherently limited to coding genes. In other words, non-coding oncogenes and tumor suppressors, if as central to cancer as for example *MYC* and *RAS*, could in principle have been discovered several decades ago.

Although the lack of non-coding genes in these early screens implies that ncRNAs are not as pivotal to tumorigenesis as their coding counterparts, it would be unwise to rule out crucial contributions based solely on this. Part of the explanation may be methodological: e.g., the number of oncogenic retroviruses is limited, and several factors may influence the appearance of cellular genes in viral genomes (Weinberg, 1982). Large-scale cancer genomics studies to date often have inherent biases toward coding genes, with e.g., resequencing efforts being directed at the coding exome. In addition, the concerted action of several dysregulated ncRNAs may together be important, even if the individual ncRNAs are incapable of cell transformation. Many of the studies that link ncRNAs to carcinogenesis are correlational in terms of clinical evidence, but they are often backed up by convincing data from cell culture and animal model systems. Finally, evidence of recurrent somatic genomic alteration of ncRNAs may be sparse compared to coding cancer genes, but it is not completely lacking. In this review, we assess available DNA-level data for ncRNAs in cancer (Table 1), and consider how the non-coding sequences that were once considered as “junk DNA” could prove to be integral players in cancer through alterations at the genomic level.

**Table 1:** **NcRNAs specifically targeted by somatic genomic alterations in cancer**.

Name	Class	Locus	Somatic alteration	Function	Cancer type	Reference
mir-15a/mir-16-1	microRNA cluster	13q14	Deletion	Tumor suppressive	CLL	Calin et al. (2002)
mir-17-92	microRNA cluster	13q31	Amplification	Oncogenic	Lymphoma	Ota et al. (2004)
LOC285194 and BC040587	lncRNA	3q13	Deletion	Tumor suppressive	Osteosarcoma	Pasic et al. (2010)
NC25	lncRNA	6q13	Mutation	Tumor suppressive	Endometrial	Perez et al. (2008)
GAS5	lncRNA/snoRNA	11q25	Gene fusion	Unknown	B-cell lymphoma	Nakamura et al. (2008)
PTENP1	Pseudogene/lncRNA	9p13	Deletion	Tumor suppressive	Colon	Poliseno et al. (2010)
SNORA42	snoRNA	1q22	Amplification	Oncogenic	Lung	Mei et al. (2012)
U50	snoRNA	6q14	Mutation	Tumor suppressive	Prostate, breast	Dong et al. (2008, 2009)

## Somatic Genomic Alteration of MicroRNAs in Cancer

MicroRNAs are small, ∼22 nucleotides long, regulatory ncRNAs that modulate gene expression by inhibitory interactions with mRNAs. They are important for a range of biological processes (Bartel, 2009), including proper development of the mammalian embryo (Bernstein et al., 2003). MicroRNAs have been the subject of intense study during the past decade, and are now showing promise as therapeutic targets and biomarkers in cancer and beyond (Rosenfeld et al., 2008; Saunders and Lim, 2009). Numerous studies have revealed frequent differential expression of microRNAs in tumors compared to normal tissues, or between clinical subtypes (Lu et al., 2005; Volinia et al., 2006). Although the overall extent to which these expression changes contribute to tumorigenesis is not clear, convincing experimental and clinical data linking dysregulation of specific microRNAs to tumorigenesis is available in several cases (recently reviewed in Lujambio and Lowe, 2012).

Likewise, genomic copy-number alterations (CNAs) in human cancers have in some cases been linked to microRNA genes, but separating non-functional passenger events from causal changes remains a challenge. One often-cited early example, based on comparative genomic hybridization array data from 227 tumors (breast, ovarian, and melanoma), showed that a high proportion of human microRNAs are located in regions of frequent CNA in cancer (Zhang et al., 2006). However, while this may partially explain why microRNAs are often deregulated in cancer, it does not necessarily implicate microRNAs in tumorigenesis. MicroRNAs are widespread across the genome and large CNAs are frequent in these tumors, and many of the alterations are therefore likely not specifically related to microRNAs. This can be exemplified by the *mir-15a*/*mir-16-1* cluster, encoded by the *DLEU2* lncRNA on chromosome 13q14, which in this study was found to be deleted in nearly 25% of both ovarian and breast cancer tumors. However, the 13q14 region harbors numerous other genes, including the *RB1* tumor suppressor, which is inactivated in many cancers including ovarian carcinoma (Cancer Genome Atlas Research Network, 2011). The contribution of *mir-15a*/*mir-16-1* is therefore not immediately obvious, and focality/size of aberrations, and/or additional functional data, needs to be taken into account to reliably pinpoint causal ncRNA genes.

In the case of *mir-15a*/*mir-16-1*, convincing data in support of a tumor suppressor role comes from chronic lymphocytic leukemia (CLL). In one of the pioneering studies that was able to link somatic alteration of microRNAs to cancer, it was shown that the 13q14 deletion, although already known to be frequent in CLL, was often restricted to a smaller 30 kb region encompassing these microRNAs (Calin et al., 2002). Later studies showed that mir-15a/mir-16-1 are proapoptotic (Cimmino et al., 2005) and, importantly, that deletion of these microRNAs in mice leads to CLL predisposition (Klein et al., 2010). However, in ovarian cancer it should be noted that *RB1*, but not the nearby *DLEU2*/*mir-15a*/*mir-16-1*, shows a clear pattern of focal deletion (Cancer Genome Atlas Research Network, 2011), suggesting that reported *mir-15a*/*mir-16-1* deletions in ovarian cancer (Zhang et al., 2006) could be passenger events. This example illustrates the difficulties involved in associating genomic aberrations with causal genes, even in the presence of convincing experimental data from other cancer types.

Some of the earliest evidence that microRNAs can have oncogenic properties come from studies of the mir-17-92 cluster on chromosome 13q31, a region known to be amplified in several cancers including diffuse large B-cell lymphoma (DLBCL; Rao et al., 1998). Although 13q31 also contains other genes, the mir-17-92 precursor was shown to be the only one at the focal center where expression correlated with copy-number amplitude in DLBCL (Ota et al., 2004). Based on a mouse model of B-cell lymphoma, it was later shown that forced expression of mir-17-92 accelerates tumor development through cooperation with c-Myc (He et al., 2005). MiR-19 was eventually pinpointed as the main oncogenic microRNA derived from this cluster, together with associated key targets in the phosphatidylinositol-3-OH kinase pathway (Olive et al., 2009; Mavrakis et al., 2010). Similarly, miR-155 causes B-cell malignancy when overexpressed in mouse B-cells (Costinean et al., 2006) while also frequently being highly expressed in human lymphomas (Eis et al., 2005), although it is unclear whether this primarily happens through transcriptional activation or genomic amplification.

Several other studies have linked CNA in cancer to microRNA genes, albeit at different levels of confidence in terms of functional significance. In one case, 16 microRNA genes were found to show correlations between expression level and copy-number amplitude in different regions frequently exposed to CNA in multiple myeloma (Lionetti et al., 2009). Frequent amplification of *mir-30d*, *mir-151*, and the C19MC microRNA cluster has been observed in hepatocellular carcinoma (Liu et al., 2011; Augello et al., 2012). Likewise, frequent copy-number gains, which correlate with gene expression, have been observed for *mir-30b* in oral squamous cell carcinomas (Shao et al., 2012). However, *mir-30b* is proximal to *MYC*, a target of amplification in numerous cancers, and the value of this category of observations is therefore unclear in the absence of data on focality or additional functional investigations.

In addition to CNA, microRNAs should in principle be susceptible to inactivation through somatic mutations, although the evidence of this has been relatively sparse so far. In one study, 15 cancer-implicated microRNAs were screened for mutations in 91 tumor cell lines, but no changes within mature microRNA sequences were identified (Diederichs and Haber, 2006). Likewise, mutation screening of 712 microRNAs in 86 ovarian tumors identified only five mutations, of which all were in different genes and only one in the mature sequence (Ryland et al., 2012). Sequencing of microRNAs that are dysregulated in CLL identified a mixture of rare (1–3 out of 75 patients) somatic and germline mutations in 8/42 microRNAs tested, including a germline substitution in *mir-16-1* in two patients, while only a few sequence abnormalities were found in 160 healthy controls (Calin et al., 2005). While the idea that microRNA mutations may be a predisposing factor in familial CLL is intriguing, more detailed genetic studies on affected families are required to better establish this. Another study based on 255 CLL patients found rare somatic mutations in the stem region of miR-16 in two cases (Ouillette et al., 2011). A somatic substitution was found in mir-518d in the central region of the stem, based on whole-genome resequencing of a single case of melanoma, but at the same time 33,344 other somatic base substitutions were identified (Pleasance et al., 2010). More comprehensive studies are needed to safely determine whether such rare mutations are functional and under selection during tumor evolution.

Non-coding RNA-associated proteins, such as those required for microRNA function and biogenesis, are also of interest in this context, and several studies show that these can be targets of somatic alteration in cancer. For example, in microsatellite instable gastric and colorectal cancers, recurrent frame-shift mutations were found in *AGO2* and *TNRC6A* (Kim et al., 2010). Likewise, recurrent somatic mutations (among other, a missense mutation) were identified in the RNase IIb domain of *DICER1*, in 30/102 non-epithelial ovarian tumors (Heravi-Moussavi et al., 2012). Although these mutations are not targeted at specific microRNAs, they provide strong support for the idea that altered ncRNA function is important for tumorigenesis.

## Genomic Alteration of LncRNAs: A Long Story Made Short

Long non-coding RNAs are broadly defined as long (arbitrarily >200 nt) transcripts that lack protein-coding capacity but otherwise often have mRNA-like properties, including multi-exonic gene structures and poly(A) tails. Studies point to diverse molecular roles, including recruitment of histone-modifying complexes to chromatin (e.g., *XIST*, *HOTAIR*; Plath et al., 2003; Rinn et al., 2007) and regulation of transcription and splicing through interactions with relevant factors (Bernard et al., 2010; Kino et al., 2010). Although early examples were described more than 20 years ago, more recent studies have revealed that mammalian genomes encode thousands of lncRNAs that are often developmentally regulated, and show weak but significant patterns of evolutionarily conservation (Ponjavic et al., 2007; Guttman et al., 2009). Their biological importance has been debated, but novel lncRNAs are now being characterized at increasing frequency, and these have been shown to have essential roles, e.g., in vertebrate development (Ulitsky et al., 2011), pluripotency (Guttman et al., 2011), and genome stability (Huarte et al., 2010).

Several studies hint at important roles in oncogenesis for lncRNAs. For example, *HOTAIR* expression is high in breast cancer tumors that are predisposed to metastasize, and its inhibition blocks metastasis in mouse models (Gupta et al., 2010), and *MALAT1* expression correlates with metastases and survival in lung cancer (Ji et al., 2003). Numerous other lncRNAs are altered in cancer at the level of gene expression (recently reviewed in Prensner and Chinnaiyan, 2011), but our knowledge is still limited when it comes to targeted genomic alterations. One recent investigation showed that two lncRNA genes on chromosome 3q13.31, *LOC285194* and *BC040587*, were frequently deleted in osteosarcoma (Pasic et al., 2010). Notably, the deletions were often highly focal, but sometimes also included the nearby protein-coding tumor suppressor *LSAMP*. The results suggest that the genes in this region, which are also coexpressed, may function as a unit. Furthermore, deletion of either lncRNA was associated with poor survival (Pasic et al., 2010). Focal deletion has also been observed in the case of a *PTEN* pseudogene, *PTENP1*, in colon cancer, which can regulate its tumor suppressive coding counterpart by competitive binding to common microRNAs (Poliseno et al., 2010). This intriguing mechanism has been further explored in cancer (Sumazin et al., 2011), and is supported by the observation that microRNAs with many targets tend to have a diluted effect on each individual target (Arvey et al., 2010). Further computational studies are needed to determine whether interactions between microRNAs and other ncRNAs are widespread and conserved (Jeggari et al., 2012).

A few studies suggest that somatic mutations in lncRNAs may be important in cancer, but this remains poorly explored. In one case, a set of 15 highly expressed and conserved lncRNAs were screened for mutations in both cancer cell lines and unmatched normal controls (Perez et al., 2008). Three lncRNAs showed consistent alterations at specific nucleotide positions in at least two cancer cell lines, although it could not be excluded that these represented rare polymorphisms. The mutations were not recapitulated in a panel of 48 matched endometrial tumors and normals, but another lncRNA, NC25, here displayed a striking pattern of somatic alteration, with mutations being present in almost half (23/48) of the patients at one of four distinct positions (Perez et al., 2008). Mutations have also been described in the 3′-end of *MALAT1* (Xu et al., 2011), a lncRNA known to be highly expressed in metastases originating from different cancers (Ji et al., 2003; Ying et al., 2012). It was also determined that the 3′-end of *MALAT1* confers the main biological activity, but the putative functional impact of the actual mutations was never evaluated (Xu et al., 2011).

Ultraconserved regions (UCRs) are genomic elements of near-perfect evolutionary conservation in multiple mammalian genomes, some of which overlap with transcribed regions (exonic, partly exonic, or intronic; Bejerano et al., 2004; Sandelin et al., 2004). UCRs are often located in cancer-associated genomic regions, and several UCRs are transcribed into non-coding transcripts (T-UCRs) whose expression is altered in cancer and is correlated with clinical subtypes and cancer-relevant cellular processes (Calin et al., 2007; Mestdagh et al., 2010). Both somatic and germline mutations have been identified in T-UCRs in colorectal cancer and CLL (Wojcik et al., 2010), but further study is needed to firmly establish if T-UCRs are specific targets of mutation in cancer, or confer heritable risk.

Long non-coding RNA have also been reported to participate in somatic gene fusions. The *GAS5* lncRNA gene, which also harbors several intronic small nucleolar RNAs (snoRNAs), has been found to fuse with the *BCL6* proto-oncogene in a patient with B-cell lymphoma (Nakamura et al., 2008). Similarly, an *ETV1* translocation to an androgen-regulated lncRNA, *PCAT-14*, has been reported in prostate cancer (Prensner and Chinnaiyan, 2011; Prensner et al., 2011). Most likely, lncRNA genes in these cases only contribute regulatory DNA to drive aberrant protein expression, and whether oncogenic lncRNAs themselves can be activated or functionally modulated through genomic translocation remains to be determined.

## SnoRNAs in Tumorigenesis

Small nucleolar RNAs constitute a well-characterized class of structural RNAs of 60–300 nucleotides in length, with roles in chemical modification of ribosomal RNAs. Emerging evidence suggests that snoRNAs may have specific roles in oncogenesis (reviewed in Williams and Farzaneh, 2012). As an example, tumor-enriched snoRNAs have been identified in lung cancer, and these are detectable in blood plasma at elevated levels in patients compared with healthy controls (Liao et al., 2010). Recent work also suggests that snoRNAs may be specific targets of somatic genomic alteration. In a study based on 10 lung cancer cell lines, it was shown that *SNORA42*, but not its protein-coding host gene, exhibits a high degree of correlation between RNA level and genomic copy-number (Mei et al., 2012). Together with functional data from loss- and gain-of-function experiments in cell lines and mouse xenograft models, this implicates an oncogenic role for *SNORA42*. However, it should also be noted that several other genes in the same genomic region (1q22) display a similar relationship between copy-number and RNA level (Li et al., 2006).

In addition to *SNORA42*, the snoRNA*U50* has been implicated in prostate and breast cancer, where it is commonly deleted together with other genes in the 6q14-22 region (Dong et al., 2008, 2009). Interestingly, identical 2-bp somatic deletions in *U50* were discovered in 9/89 of prostate and 4/49 breast tumors studied, and germline homozygosity for the same deletion was associated with prostate cancer in a larger case-control study (Dong et al., 2008, 2009). Collectively, these studies are intriguing and point to specific roles for snoRNAs in cancer, beyond housekeeping functions related to protein synthesis.

## Concluding Remarks and Perspectives

Here, we have attempted to summarize current evidence for specific somatic alteration of ncRNAs in cancer (Table 1). Though only briefly discussed here, germ line mutations are also of paramount importance: in addition to potential benefits for early cancer diagnostics, associations between natural genetic variants, or familial mutations, and cancer susceptibility can pinpoint important cancer ncRNAs. In addition, only when considering both genetic and epigenetic aspects will the picture be complete.

It is already clear that altered ncRNA function, by genomic change or other means, is of importance in tumorigenesis. However, due to the rapid evolution of high-throughput genomics, the detailed map of ncRNA alterations in cancer is likely to change significantly in the near future. In particular, resequencing in cancer is transitioning from whole-exome to whole-genome (Meyerson et al., 2010), effectively putting more emphasis on non-coding sequences. This technique is not without challenges, including the separation of low-frequency functional alterations from numerous non-contributing ones (Boehm and Hahn, 2011). However, increasingly comprehensive patient cohorts will eventually help reveal whether specific somatic alteration of ncRNAs is commonplace in cancer. In addition to improving our understanding of tumor development, these studies hold the exciting promise of pinpointing important novel ncRNAs that are crucial to the physiology of the normal mammalian cell.

## Conflict of Interest Statement

The authors declare that the research was conducted in the absence of any commercial or financial relationships that could be construed as a potential conflict of interest.
